# Protein levels alter yak rumen microbiota profiles, meat properties, and *longissimus dorsi* metabolites

**DOI:** 10.5713/ab.25.0027

**Published:** 2025-07-11

**Authors:** Jiyuan Zhang, Shuxiang Wang, Shatuo Chai, Shengchun Xu, Ziming Zeng, Zhilong Wang, Xun Wang, Yingkui Yang, Shujie Liu, Jiaying Lv, Mingliang Wang, Xinjun Zhang

**Affiliations:** 1 Qinghai Academy of Animal Husbandry and Veterinary Sciences in Qinghai University, Xining, China; 2 Key Laboratory of Plateau Grazing Animal Nutrition and Feed Science of Qinghai Province, Xining, China; 3 Yak Engineering Technology Research Centre of Qinghai Province, Xining, China; 4 Tibet Vocational and Technical College, Xizang, China; 5 Huangzhong District Xibao Town Ecological Dairy Farming Base of Qinghai Province, Xining, China; 6 Qinghai Xia Hua Halal Meat Products Co., Ltd., Haiyan, China

**Keywords:** Dietary Protein Levels, Growth Performance, Meat Quality, Muscle Metabolome, Rumen Microbiota, Yak

## Abstract

**Objective:**

The study investigated how varying protein levels in low-energy diets affected the microbiota, meat quality, and metabolomics of the *longissimus dorsi* muscle in yaks. The aim was to determine the optimal yak diet for growth and meat quality under low-energy conditions.

**Methods:**

Twenty-four adult male yaks were divided into two groups of 12: the low-energy, medium-protein (LM) group and the low-energy, high-protein (LH) group. The study analysed rumen microbiota and *longissimus dorsi* muscle metabolites using 16S rDNA gene sequencing and untargeted metabolomic analysis. The effects of the diets on growth performance, meat quality and microbial community composition were evaluated.

**Results:**

There were no significant differences in growth performance between the LH and LM groups. However, the LH group had a lower pH value at 45 minutes after death and was better for meat colour and tenderness. There were no significant differences in average daily gain, cooking loss, hardness, elasticity, adhesiveness, chewiness, or the pH at 24 hours after death in the *longissimus dorsi* muscle between the groups. Microbial community analysis revealed no significant differences in diversity indices; however, it did indicate distinct bacterial composition between the groups. Predictions of function suggested the LM group had a higher level of enrichment and a greater number of unique operational taxonomic units compared to the LH group. Metabolomic analysis revealed differences in muscle metabolites and metabolic pathways, with the LM group having a higher capacity for fatty acid and selenocompound metabolism, implying greater energy utilisation efficiency and antioxidant function.

**Conclusion:**

The study suggests that a diet with 14% protein, as part of low-energy diets, is best for increasing yak fattening. This is because it improves energy use and antioxidant function, without affecting growth.

## INTRODUCTION

Yaks are economically significant animals in and around the Tibetan Plateau, particularly in the lives of farmers and herders in the highland pastoral areas [1]. In recent years, global climate change and increasing environmental pressures have posed significant challenges to pasture production and quality on the Tibetan Plateau [2]. The combination of low temperatures and high altitude results in elevated energy expenditure and protein requirements for yaks. The pasture resources on the Tibetan Plateau are typically scarce in terms of both abundance and nutritional quality, exhibiting considerable seasonal fluctuations. This results in notable discrepancies in the protein requirements of yaks at different growth stages and seasons [3]. In order to enhance yak performance and meat quality, the optimisation of feeding conditions and nutritional supply represents a pivotal concern [4]. As a vital nutrient for yak growth, development, immune function and metabolism, the level of protein supply has a direct impact on growth performance, rumen microbial community structure and meat quality [5]. In this context, this study integrates 16S rDNA sequencing, meat quality assessment, and non-targeted muscle metabolomics to investigate how dietary protein gradients reshape rumen microbial architecture and coordinate host-muscle metabolic reprogramming in plateau yaks under chronic hypoxic and seasonal nutritional stress, establishing a multi-omics framework that deciphers the energy-nitrogen coupling mechanisms governing microbial-host crosstalk for optimizing protein utilization efficiency and precision feeding systems in alpine ruminant production. This will enhance the efficiency of yak breeding, improve the meat quality and promote the sustainable development of the yak industry.

## MATERIALS AND METHODS

### Experimental design

The Animal Experiment Committee of Qinghai University (QHU2020067) approved the use of experimental animals. In this study, prior to experimental initiation, 24 healthy adult male yaks (aged 2.5–3 years) with comparable body weights (198±10 kg, mean±standard deviation) were selected from a centralized feeding facility and randomly allocated into two groups: the low-energy medium-protein group (LM) and the low-energy high-protein group (LH), with 12 yaks per group. A single-factor completely randomized block design was employed, with both groups receiving an energy level of 9.5 MJ/kg. Dietary energy and protein levels were referred to Hao et al [6]. The LM group received a protein level of 14%, while the LH group received a protein level of 16%.

The experiment was conducted at the standardized cattle and sheep breeding demonstration farm in Jinyintan, Haiyan County, Qinghai Province. Prior to the experiment, the yaks were dewormed and tagged. Each group was housed in a separate pen and had *ad libitum* access to water. Feeding was conducted with consistent timing, quantities, and personnel. Diets were formulated and mixed according to specified compositions, and feed was provided twice daily. The pre-trial period lasted 10 days, followed by a formal experimental period of 180 days. On the 90th day of the experiment, 500 g of feed samples were collected from each of the barns of the LH and LM groups, and 100 g of the subsamples were determined in the laboratory for crude protein (CP), neutral detergent fiber (NDF), acid detergent fiber (ADF), calcium (Ca) and phosphorus (P). The ME value was also calculated. An additional 100 g sample was taken and dried in a forced air oven (Model WHL-25AB; TAISITE) at 60°C for 48 h and then ground in a pulverizer (Model FW100; TAISITE). The samples were analyzed after passing through a 1 mm sieve. CP, Ca and P contents were determined according to AOAC official method 990.03 [7]. NDF and ADF were measured following Van Soest et al [8]. The detailed composition and nutritional levels of the experimental diet are presented in Table 1 (in dry matter [DM]).

### Yak *longissimus dorsi* muscle samples

Following acquisition of the Animal Quarantine Certificate from local authorities, all experimental yaks were humanely managed in accordance with GB/T 19477-2004 (Cattle Slaughter Operating Procedures). A 24-hour pre-slaughter fasting period with *ad libitum* water access was implemented to comply with animal welfare guidelines. Post-feeding phase termination, 24 yaks were humanely euthanized through standardized captive bolt stunning and exsanguination protocols. Within 15 minutes post-mortem, 200 mg tissue specimens from the *longissimus dorsi* muscle were aseptically collected using sterilized biopsy instruments. Randomization procedures yielded five representative samples per experimental group for untargeted metabolomic profiling. Muscle samples underwent sequential preservation protocols: primary stabilization at 0°C–4°C for 24 hours (postmortem aging phase), followed by dual preservation pathways—subsamples for metabolomic analysis were snap-frozen in liquid nitrogen-chilled cryovials (Nunc) and transferred to −80°C ultra-low temperature storage, while archival specimens were vacuum-sealed and maintained at −20°C for long-term preservation.

### Measurement of pH, colour and shear

On day 180 of the trial (March 1, 2023), five yaks were randomly selected from each group (LH and LM), for a total of 10 yaks. Rumen fluid was collected prior to morning feeding using a sterile oral stomach tube sampler. The initial 100 mL of rumen fluid was discarded to minimize contamination from saliva. Subsequently, 150 mL of fluid was collected and immediately filtered through four layers of sterile cheesecloth (pore size: 250 μm) to remove coarse particulate matter. The pH of the rumen fluid was measured immediately with a bench-top acidimeter (Model HI221; HANNA) To ensure the accuracy of the results, at least three determinations were made for each sample, with the average value taken as the final result. Following the excision of the samples, they were permitted to rest at room temperature for a period of 40 minutes. A portable colorimeter (Model DS-100; CAIPU) was employed to ascertain the lightness (L*), redness (a*) and yellowness (b*) values. To ensure accuracy, each sample was measured twice. The determinations were made using a Warner-Bratzler shearometer, with a minimum of three shear tests conducted on each sample in order to obtain consistent and reliable data.

### Cooking loss measurement

The meat sample was initially weighed (m1) and sealed in a cooking bag. The sealed sample was subsequently heated in a water bath maintained at 85°C for 30 minutes, followed by cooling to room temperature. After cooling, the sample was reweighed (m2). Cooking loss (%) was calculated according to the Eq. 1:


(1) Cooking loss (%)=100×([m1-m2]/m1).

### Texture measurement

After cooking, the meat samples were cooled to room temperature and cut into small cubes with a volume of 1,000 mm^3^. Texture parameters were measured using a texture analyzer (Model HP-YSD609; Henpina), with each sample measured five times, and the average value was recorded. The instrument settings were as follows: P5 (TA-44) stainless steel cylindrical probe, pre-test speed of 2.0 mm/s, test speed of 1.0 mm/s, post-test speed of 1.5 mm/s, compression ratio of 50%, 5-second interval between two compressions, and the start mode set to auto-20 g.

### 16S rDNA sequencing of rumen fluid samples

The collected rumen fluid samples were rapidly frozen in liquid nitrogen and subsequently stored at −80°C for determination of 16S rDNA of rumen fluid microorganisms. The samples were subsequently thawed on ice and mixed thoroughly, after which the microbial DNA in the rumen fluid samples was extracted using the CTAB (Cetyltrimethylammonium Bromide) method [9]. The V3-4 hypervariable region of bacterial 16S rRNA gene were amplified with the universal primer 338F (5′-ACTCCTACGGGAGGCAGCAG-3′) and 806R (5′-GGACTACNNGGGTATCTAAT-3′). For each sample, 8-digit barcode sequence was added to the 5′ end of the forward and reverse primers (Allwegene). The polymerase chain reaction (PCR) was carried out on a Mastercycler Gradient (Eppendorf) using 25 μL reaction volumes, containing 12.5 μL 2× Taq PCR MasterMix (Vazyme Biotech), 3 μL BSA (2 ng/μL), 1 μL Forward Primer (5 μM), 1 μL Reverse Primer (5 μM), 2 μL template DNA, and 5.5 μL ddH2O. Cycling parameters were 95°C for 5 min, followed by 28 cycles of 95°C for 45 s, 55°C for 50 s and 72°C for 45 s with a final extension at 72°C for 10 min. The PCR products were purified using a Agencourt AMPure XP Kit (Beckman Coulter). Sequencing libraries were generated using NEB Next Ultra II DNA Library Prep Kit (New England Biolabs) following the manufacturer’s recommendations. The library quality was assessed by Nanodrop 2000 (Thermo Fisher Scientific), Agilent 2100 Bioanalyzer (Agilent Technologies), and ABI StepOnePlus Real Time PCR System (Applied Biosystems), successively. Deep sequencing was performed on Illumina Miseq (Illumina) platform at Beijing Allwegene Technology. After the run, image analysis, base calling and error estimation were performed using Illumina Analysis Pipeline Version 2.6 (Illumina).

Paired-end sequencing data (2×250 bp) generated from the Illumina MiSeq platform were processed through a standardized bioinformatics pipeline. Initial quality control was performed using Trimmomatic (v0.36) with sliding-window trimming (50 bp window, Q20 threshold) and PEAR (v0.9.6) for ambiguous base (N) removal and read merging (minimum overlap: 10 bp; mismatch rate≤0.1). The processed sequences (raw_tags) were subsequently subjected to a dual-strategy chimera filtering approach. This approach entailed reference-based detection against the SILVA 132 database and de novo identification via VSEARCH (v2.7.1), yielding high-quality clean_tags (>150 bp). Operational taxonomic units (OTUs) were then clustered at 97% sequence similarity using QIIME (v1.8.0) with the UPARSE algorithm [10]. This was followed by taxonomic classification against the Greengenes database (v13_8) and singleton removal (abundance≤3). Microbial diversity analyses included α-diversity metrics (Chao1, Shannon, PD-whole-tree) calculated from rarefied OTU tables (10,000 reads/sample) and β-diversity assessment via unweighted UniFrac distances visualized through principal coordinate analysis. Microbiota functional potential was predicted with PICRUSt2, and the outputs were annotated against the Kyoto Encyclopedia of Genes and Genomes (KEGG) database (Release 99.0) for KO, pathway, and EC classification.The amplicon sequencing data have been uploaded to the NCBI database and are publicly accessible. The BioProject accession ID is PRJNA1188369.

### Metabolomic analysis of yak *longissimus dorsi* muscle

200 mg tissue specimens from the *longissimus dorsi* muscle were aseptically collected using sterilized biopsy instruments. The *longissimus dorsi* muscle of yaks was subjected to analysis using liquid chromatography–mass spectrometry (LC–MS). The LC-MS analysis was carried out on a Thermo UHPLC-Q Exactive HF-X system, fitted with an ACQUITY HSS T3 column (100 mm×2.1 mm i.d., 1.8 μm; Waters). The mobile phase comprised 0.1% formic acid in water (95:5, v/v) (solvent A) and 0.1% formic acid in a mixture of acetonitrile and isopropanol (47.5:47.5:5, v/v) (solvent B). The flow rate was maintained at 0.40 mL/min, with the column temperature kept at 40°C. Samples were ionised via electrospray, and mass spectra were recorded in both positive and negative ion scanning modes. The raw LC/MS data were processed using Progenesis QI software (Waters), and a three-dimensional data matrix in CSV format was exported. The data matrix contained information such as sample details, metabolite names, and mass spectral response intensity. Peaks corresponding to internal standards as well as known false-positive peaks (such as noise, column bleed, and derivatised reagent peaks) were excluded, and the data were de-redundant and pooled.

### Data processing

Statistical analyses were performed using R software (v4.1.0, http://www.r-project.org/) and SPSS26.0. The community diversity index was obtained based on 16S rRNA gene amplicon sequencing data. The species richness of the rumen microbial community was estimated using the Chao1 and Sob indices, while the α-diversity (species number and evenness) was obtained using the Shannon and Simpson indices. The β-diversity was analysed and visualised using non-metric multidimensional scaling analysis based on Bray–Curtis differences. The likelihood ratio test in the “Edge R” package detects differences in the relative abundances of OTUs between treatments and adjusts the p-value for the false discovery rate (FDR) [11]. Partial least squares discriminant analysis (PLS-DA) was used to detect the different compounds in the Metabolites of bovine *longissimus dorsi* muscle. Metabolic enrichment and pathway analyses were based on the KEGG database ( http://www.genome.jp/keg/).

## RESULTS

### Effects of diets with different protein levels on growth performance and slaughter traits of yaks

The study revealed that there was no statistically significant difference (p>0.05) between the LH and LM groups in terms of average daily weight gain, carcass weight, and slaughter rate. While the average daily weight gain of yaks in the LM group exhibited a marginal increase compared to the LH group, this discrepancy did not attain a statistically significant level. This finding indicates that alterations in dietary protein levels did not exert a substantial influence on yak growth performance under the experimental conditions employed (Table 2).

### Effects of diets with different protein levels on ph, color, shear force, and cooking loss of yak *longissimus dorsi* muscle

Dietary protein levels had significant effects on the pH at 45 minutes and L*, a*, and b* values of the yak *longissimus dorsi* muscle (p<0.05, Table 3). Comparing the LH and LM groups, it was found that the pH at 45 minutes in the LH group was significantly lower than that in the LM group (p<0.01, Table 3). There was no significant difference in the pH at 24 hours between the LH and LM groups (p>0.05, Table 3). The L*, a*, and b* values of the *longissimus dorsi* muscle were significantly higher in the LH group compared to the LM group (p< 0.01). The shear force of the *longissimus dorsi* muscle was significantly lower in the LH group compared to the LM group (p<0.01, Table 3). No significant difference in cooking loss was observed between the two groups (p>0.05, Table 3).

### Effects of diets with different protein levels on the texture characteristics of yak *longissimus dorsi* muscle

Statistically significant differences in texture parameters (hardness, elasticity, cohesiveness, or chewiness) were not observed between the LH and LM groups (p>0.05; Table 4).

### Differences in rumen microbiome of yaks fed diets with different protein levels

**Figure 1:**
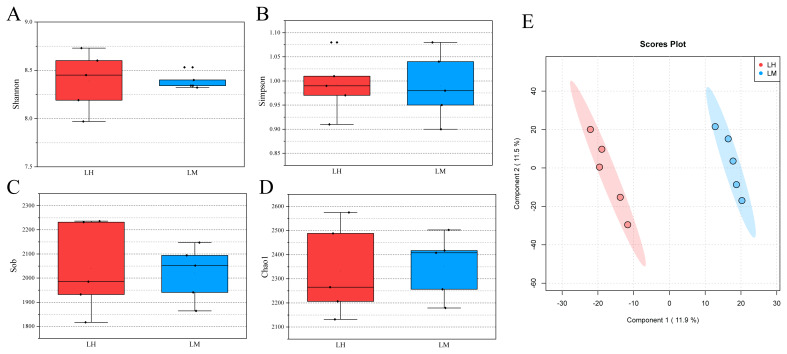
Differences in rumen microbial diversity of yaks. (A) The difference in Shannon index between the LH group and the LM group (p>0.05). (B) The difference in Simpson’s index between the LH group and the LM group (p>0.05). (C) The difference in Sob index between the LH group and the LM group (p>0.05). (D) The difference in Chao1 index between the LH group and the LM group (p>0.05). (E) Partial least squares discriminant analysis (PLS-DA) was used to compare OTUs between the LH group and the LM group. The results showed significant differences between the two groups (p<0.05). LH, low-energy high-protein; LM, low-energy medium-protein; OTU, operational taxonomic unit.

**Figure 2:**
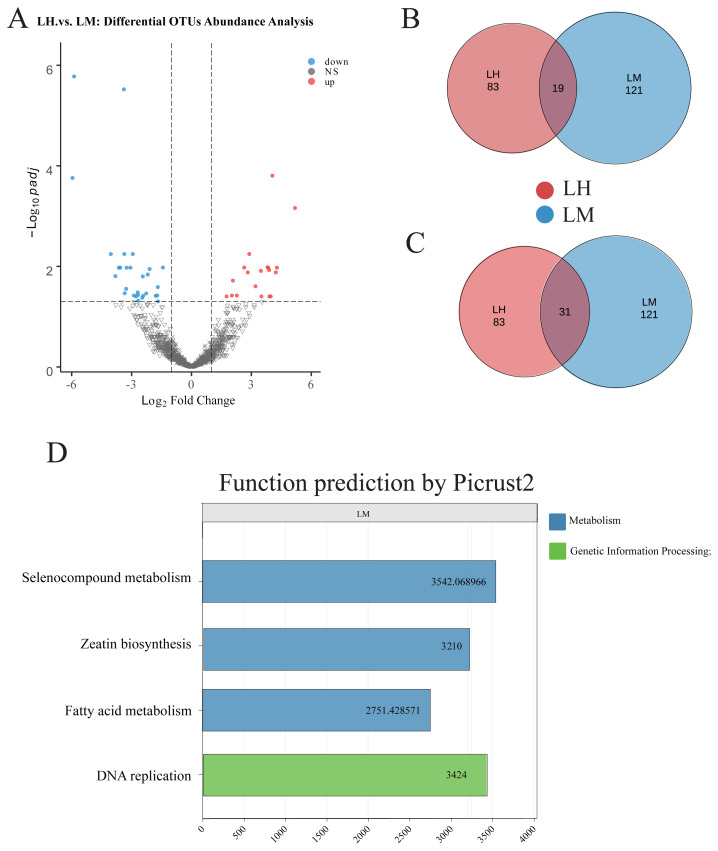
Screening of OTUs and functional enrichment analysis of rumen microorganisms between the LH and LM groups. (A) Volcano plot of OTU differences between LH and LM, set with p-value (FDR)<0.05 and |log_2_(fold change)|>1. In the figure, the solid circles on the right represent OTUs significantly enriched in LH, while those on the left represent OTUs significantly enriched in LM. (B, C) The non-overlapping regions represent unique OTUs for each group, while the overlapping regions indicate OTUs that are significantly enriched in either LH or LM. (D) Functional prediction was performed using Picrust2 for OTUs significantly enriched in the LM group and OTUs unique to the LM group, and functional categories with higher abundance were selected through screening. LH, low-energy high-protein group; LM, low-energy medium-protein group; OTU, operational taxonomic unit; FDR, false discovery rate.

There were no significant differences in the Shannon index (Figure 1A), Simpson index (Figure 1B), Sob index (Figure 1C), or Chao1 index (Figure 1D) of the rumen microbiome, indicating that low-energy high-protein and medium-protein diets had no significant effect on the α-diversity of the rumen microbiome. However, PLS-DA analysis showed significant clustering of rumen microbiota composition between the LH (low-energy, high-protein diet) and LM (low-energy, medium-protein diet) groups, suggesting that dietary protein level had a significant effect on microbial community structure (Figure 1E). Consequently, we performed differential enrichment analysis of OTUs between the LH and LM groups using Wekemo Bioincloud ( https://www.bioincloud.tech) (Figure 2A). Specifically, the DESeq2 algorithm (based on a negative binomial distribution model) was applied to compare OTU relative abundances between groups, with significance defined as a **Benjamini-Hochberg-corrected p-value (FDR)<0.05** and **|log_2_(fold change)|>1**. Volcano plots were generated using the platform’s built-in ggplot2 toolkit to visualize differentially abundant OTUs (Figure 2A). There were 19 significantly enriched OTUs and 83 unique OTUs in the LH group compared with the LM group (Figure 2B). In contrast, the LM group exhibited 31 significantly enriched OTUs and 122 unique OTUs compared to the LH group (Figure 2C). Functional prediction of the significantly enriched and unique OTUs in the LM group revealed enhanced functions related to DNA replication, fatty acid metabolism, selenocompound metabolism, and zeatin biosynthesis compared to the LH group.

### Metabolomic analysis of yak *longissimus dorsi* muscle

**Figure 3:**
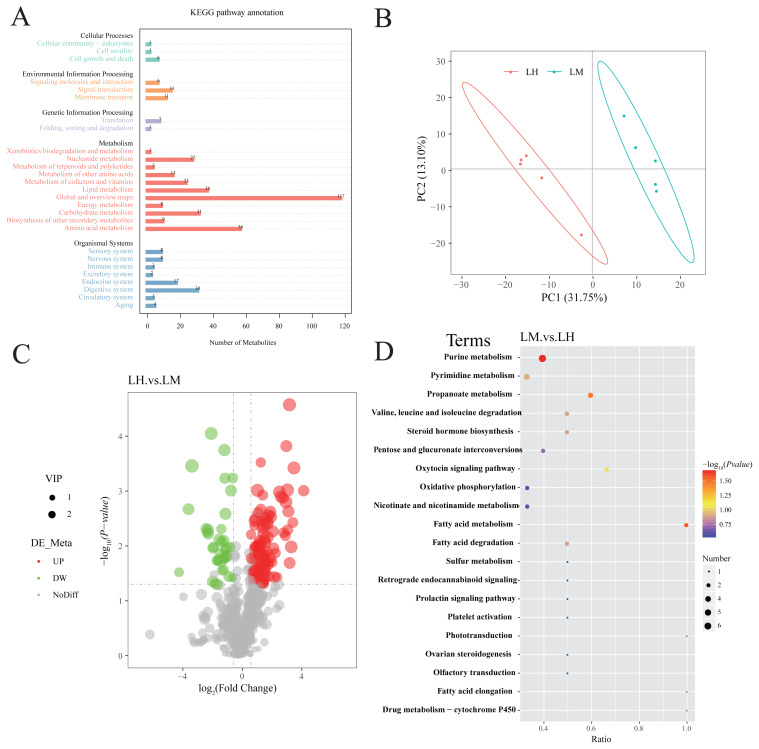
Screening of differential metabolites and metabolic pathway enrichment in the *longissimus dorsi* between the LH and LM groups based on PLS-DA analysis. (A) KEGG annotation of metabolites in the LM and LH groups revealed that 8 metabolites were involved in cellular processes, 31 were involved in environmental information processing, 8 were involved in genetic information processing, 326 were involved in metabolic processes, and 75 were involved in organism systems. (B) Pairwise least squares discriminant analysis (PLS-DA) was used to compare the *longissimus dorsi* metabolites of the LH group and the LM group. The results showed significant differences between the two groups (p<0.05). (C) Based on the PLS-DA analysis results, a volcano plot was drawn using differential metabolites with VIP>1 and p<0.05. A total of 279 differentially expressed metabolites were identified. Among these, 139 metabolites were upregulated in the LH group compared to the LM group and are marked with solid circles on the right. Additionally, 140 metabolites were upregulated in the LM group compared to the LH group and are marked with solid circles on the left. (D) Metabolic pathway bubble diagram for the LH and LM groups. In the diagram, each bubble represents a metabolic pathway. The larger the bubble, the greater the influencing factor. The darker the bubble color (closer to red), the more significant the enrichment. KEGG, Kyoto Encyclopedia of Genes and Genomes; LH, low-energy high-protein group; LM, low-energy medium-protein group.

LC-MS analysis of metabolites from two groups of LH (low-energy, high-protein diet) and LM (low-energy, medium-protein diet) yak *longissimus dorsi* muscles with different protein content followed by data preprocessing. The preprocessing steps included filtering low-quality peaks, filling in missing values, normalization, QC sample RSD assessment, and data transformation. After database searching, 422 cationic metabolites and 363 anionic metabolites were identified. KEGG annotation (Figure 3A) revealed that 8 metabolites were involved in Cellular Processes, 31 in Environmental Information Processing, 8 in Genetic Information Processing, 326 in Metabolism, and 75 in Organismal Systems. PLS-DA analysis of metabolites from the LH and LM groups showed significant differences between the two groups (R2Y: 0.99, Q2Y: 0.60, F: 153.03, p: 0.009 [p<0.01]; Figure 3B). Based on the PLS-DA results, differential metabolites were selected using VIP>1 and p<0.05. A total of 279 differential metabolites were identified, with 139 upregulated and 140 downregulated in the LH group compared to the LM group (Figure 3C).

To evaluate the effects of medium- and high-protein diets on the *longissimus dorsi* muscle metabolites, KEGG annotation was performed on metabolites significantly upregulated in the LM group relative to the LH group (Figure 3D). Significant differences between the groups were observed in purine metabolism, fatty acid metabolism, and propanoate metabolism pathways (p<0.05). Based on KEGG pathway identification, six metabolites—uric acid, xanthosine, guanosine monophosphate (GMP), ADP-ribose, 2′-deoxyadenosine, and guanosine—were mapped to the purine metabolism pathway. Two metabolites—palmitic acid and L-palmitoylcarnitine—were mapped to the fatty acid metabolism pathway, while three metabolites—methylmalonic acid, succinic acid, and methylmalonate—were mapped to the propanoate metabolism pathway. These 11 metabolites were identified as characteristic metabolites. These metabolic processes and signaling pathways play crucial roles in maintaining normal physiological functions, growth and development, energy balance, immune response, and neural transmission, which may be closely related to the higher average daily gain (ADG) observed in the medium-protein group.

### Correlation between metabolomics and rumen microbiota

**Figure 4:**
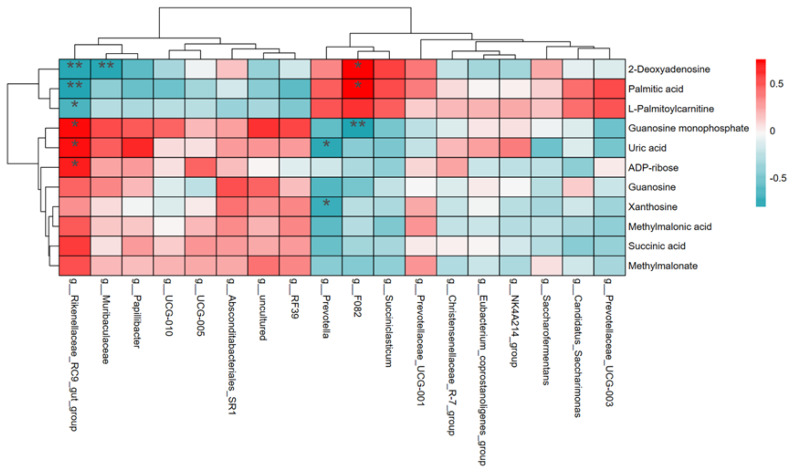
Rumen microorganisms and *longissimus dorsi* metabolites based on Spearman’s correlation analysis. * p<0.05, ** p<0.01 indicate statistically significant correlations between rumen microbiota and metabolites.

Figure 4 displays the correlation analysis between ruminal microbiota and metabolites, visualized through a heatmap based on Spearman correlation analysis with FDR-adjusted (only correlations with an absolute ρ-value>0.5 were considered significant). This analysis was performed using the Wekemo BioCloud platform (https://www.bioincloud.tech). The findings suggest that the dynamic variations of certain metabolites are strongly linked to the abundance of specific microbial groups. Rikenellaceae_RC9_gut_group was positively correlated with ADP-ribose, uric acid, and GMP, but negatively correlated with L-palmitoylcarnitine, palmitic acid, and 2′-deoxyadenosine. Muribaculaceae showed a negative correlation with 2′-deoxyadenosine. Prevotella was negatively correlated with uric acid and xanthosine. F082 was positively correlated with palmitic acid and 2′-deoxyadenosine, but negatively correlated with GMP.

## DISCUSSION

There was no significant difference in production performance between the LH and LM groups. This finding is consistent with the results reported by Boonsaen et al [12], who examined the effects of dietary protein and energy sources on the performance, carcass quality, and production costs of feedlot Kamphaeng Saen cattle. The investigation revealed that diets with varying CP levels (12% CP and 14% CP) exerted no statistically significant influence (p>0.05) on feedlot performance, carcass characteristics, and meat quality. However, Kamphaeng Saen cows fed a 12% CP concentrate of forage-based total mixed rations (cTMR) demonstrated profitability. Similarly, Li et al [13] found that the growth rate and final carcass weight of F1 Angus×Chinese Xiangxi yellow cattle were not influenced by dietary energy and protein levels or sex, indicating that different protein levels had no significant impact on ADG or slaughter performance in this crossbreed.

This phenomenon can be explained by the physiological characteristics of protein metabolism in ruminants. Rumen microbiota can efficiently utilize non-protein nitrogen in the diet to synthesize microbial protein, meeting the growth and production needs of ruminants [14]. Excess protein may exceed the rumen microbiotas’ utilization capacity, leading to protein wastage [15]. In the late growth phase of yaks, the reduced growth rate decreases protein requirements, and excess protein is excreted via the urea cycle, increasing unnecessary energy expenditure. This observation aligns with the findings of Kim et al [16], who reported that increased protein levels did not further enhance the ADG of beef cattle under moderate and low stress conditions. A protein level of 14% was sufficient to meet the growth requirements of yaks, and increasing protein levels beyond this not only failed to significantly improve weight gain but also potentially increased the metabolic burden [17].

The richness and diversity of the rumen microbiota are crucial for the digestive metabolism of ruminants. Microorganisms in the rumen ferment dietary fiber and non-structural carbohydrates, producing volatile fatty acids (VFA), which serve as the primary energy source for the animal [18]. A meticulous examination of the rumen microorganisms (α-diversity index) reveals no statistically significant disparities between the LH and LM groups. Although no significant differences in α-diversity were observed between the LH and LM groups, β-diversity analysis revealed significant differences in microbial community composition between the two groups, consistent with previous research findings [19]. This finding indicates that, while diets with varying protein levels did not result in substantial differences in rumen microbial α-diversity (p>0.05), microbial community composition, as assessed by β-diversity analyses, exhibited statistically significant variations (p<0.05). This suggests that dietary protein levels primarily influenced the remodeling of microbial community structure rather than the alteration of overall diversity. It is noteworthy that alterations in community structure may carry more biological significance than α-diversity indicators alone.

The structural stability of rumen microbial diversity (as evidenced by α-diversity indices) coupled with significant β-diversity shifts between dietary groups, implies that protein level modulation induced microbial community restructuring rather than altering overall species richness. This phenomenon aligns with established microbial ecological principles where environmental pressures preferentially select specific functional taxa without necessarily diminishing total diversity [20].

The higher number of unique OTUs in the LM group compared to the LH group may indicate that certain microbial taxa are better adapted to moderate protein environments and perform specific metabolic functions under these dietary conditions. Diether and Willing [21] scholars explored the role of gut microbial fermentation of dietary proteins and how this fermentation affects host health and metabolism. Their findings revealed that diverse protein diets prompt rapid alterations in the composition and metabolites of microbial communities, with certain microbial taxa demonstrating distinct adaptations and specialized metabolic functions in different protein environments.

Functional predictions of the differential microbiotas revealed that, compared to the LH group, the LM group was significantly enriched in several metabolic pathways and functions, including DNA replication, fatty acid metabolism, selenocompound metabolism, and zeatin biosynthesis. The enrichment of fatty acid metabolism in the LM group may affect microbial community energy storage and cell membrane synthesis. Fatty acids are essential for the structure and function of microbial cells, and enhanced fatty acid metabolism contributes to increased membrane fluidity and stability, thereby improving cell viability and facilitating cell signaling and immune functions [22]. Liu et al [23] found that BCVFA supplementation increased ADG and feed conversion in dairy cows, and that DM digestibility, organic matter, CP, ether extract, and NDF and ADF increased linearly with increasing levels of BCVFA supplementation. Such changes may also affect the dynamics of rumen microbiotas, which in turn may influence energy uptake and nutrient absorption by the host. Such changes may also influence the dynamics of the rumen microbiota, thereby affecting the host’s energy intake and nutrient absorption. Furthermore, the enrichment of selenocompound metabolism is particularly notable, as selenium is a crucial trace element involved in antioxidant and immune regulation. Zhang et al [24] found that selenium supplementation improved the antioxidant status of dairy cows, enhanced membrane fluidity and stability, and increased cell viability. The enhanced selenocompound metabolism observed in the LM group may provide additional health benefits to the host by improving its ability to cope with oxidative stress.

The functional prediction of rumen microbiota revealed the presence of a zeatin biosynthesis pathway. Notably, zeatin, a cytokinin hormone classically characterized in plants, raises questions about its biological relevance in the yak rumen ecosystem. A possible explanation for this paradox could lie in the limited specificity of homology-based annotations. For instance, bacterial isoprenyltransferases and methyltransferases may share conserved structural domains (PF09265) with the plant LOG enzyme involved in cytokinin activation, yet their actual catalytic functions could differ due to divergent substrate specificity. Additionally, horizontal gene transfer from plant-associated microbes (endophytes) to rumen bacteria, potentially facilitated by prolonged exposure to plant-rich diets, remains a hypothetical mechanism that requires phylogenetic evidence. Residual plant-derived DNA from corn-based feedstuffs might also introduce exogenous sequences into metagenomic datasets, necessitating stringent bioinformatic filtering to distinguish microbial genes from dietary contaminants. From an ecological perspective, the potential microbial production of zeatin-like metabolites warrants validation, as such compounds could hypothetically act as signaling molecules influencing host-microbe crosstalk. Future studies integrating metatranscriptomics to assess pathway activity and *in vitro* assays using bacterial isolates are critical to confirm whether this pathway is functionally active in rumen microbiota or represents an artifact of prediction algorithms.

The LM group was predominantly enriched with bacteria related to fatty acid metabolism, contrasting with the rumen microbiota of the LH group, which was more oriented towards protein metabolism. This discrepancy may result in inefficient protein utilization, leading to no further weight gain despite higher protein intake. Although different dietary protein levels did not significantly alter the diversity of yak rumen microbiota, they led to distinct microbial compositions and unique functional characteristics. Dietary protein levels do not significantly alter the diversity of the yak rumen microbiota, but they do lead to distinct microbial compositions and unique functional characteristics. The diversity and structure of the rumen microbiota are crucial for the host’s digestion and energy intake, and different protein levels in the diet may impact the composition and function of the microbial community [25].The LM diet fostered a microbial community with higher metabolic potential for health and nutrient metabolism, which has important implications for optimizing yak feed formulation.

This study demonstrated a close relationship between the rumen microbiota and the metabolites of the yak *longissimus dorsi* muscle through correlation analysis. Metabolomic analysis revealed significant differences in metabolite profiles between the LH and LM groups, particularly in fatty acid, purine, and propanoate metabolism pathways. Correlation analysis showed significant associations between specific microbial communities and particular metabolic compounds. Notably, key genera such as Rikenellaceae_RC9_gut_group, Muribaculaceae, Prevotella, and F082 were significantly correlated with several metabolites involved in energy and nucleotide metabolism.

The rumen microbiota is a crucial component of digestive tract function and metabolic activity in animals. The abundance of Rikenellaceae_RC9_gut_group was significantly higher in the LH group compared to the LM group (p<0.05), as shown in Supplement 2. This increase may be related to the nitrogen-rich high-protein diet.This group is known for its ability to degrade complex polysaccharides and proteins. Its positive correlation with ADP-ribose, uric acid, and GMP suggests that it can enhance rumen nucleotide metabolism. However, it was negatively correlated with L-palmitoylcarnitine and palmitic acid, which are part of the fatty acid metabolism pathway. L-palmitoylcarnitine plays a critical role in fatty acid metabolism by facilitating the transport of long-chain fatty acids, such as palmitic acid, into mitochondria, thereby promoting fatty acid oxidation and energy production [26]. A deficiency of L-palmitoylcarnitine and long-chain fatty acids may hinder fatty acid metabolism, resulting in stunted growth, weight loss, and impaired immune function [27]. Excessive purine metabolism can disrupt fatty acid metabolism through oxidative stress, insulin resistance, and inflammatory responses, ultimately affecting cell health and overall metabolic balance. Rikenellaceae_RC9_gut_group may play an antagonistic role in fatty acid metabolism. Fatty acid metabolism pathways were more active in the LM group compared to the LH group.

The significant increase in Prevotella abundance in the LM group (p<0.01) is particularly noteworthy (Supplement 2). This genus facilitates cellulose fermentation by degrading complex carbohydrates, thereby improving feed conversion and animal productivity [28]. The moderate protein level in the LM group optimized carbon metabolism, producing more VFA and enhancing energy supply and rumen metabolic efficiency. Increased Prevotella abundance may reduce levels of uric acid and xanthosine, which leads to decreased purine metabolism, thereby improving the efficiency of fatty acid and propanoate metabolism by reducing oxidative stress, enhancing insulin sensitivity, and reducing inflammation [29]. The negative correlation between Prevotella and uric acid and xanthosine suggests that the reduction of these metabolites in the LM group may have liberated energy metabolism pathways, enhancing the activity of fatty acid and propanoate metabolism.

F082 is a bacterium involved in cellulose degradation and short-chain fatty acid production, playing a crucial metabolic role in the rumen. Xu et al [30] found that rumen microorganisms, including F082 bacterium, were enriched in the rumen of high ADG heifers, potentially promoting carbohydrate degradation and increasing the production of VFA, such as butyrate, thereby contributing to improved ADG. Although the abundance of F082 in the LM group was only slightly higher than in the LH group, with no significant difference (p>0.05; Supplement 2). Its correlation with key metabolites suggests potential metabolic advantages in the LM group. F082 showed a significant positive correlation with palmitic acid in the LM group, which may enhance fatty acid metabolism by promoting palmitic acid production, thereby providing more energy reserves and structural support for yaks, optimizing energy utilization. Additionally, F082 was negatively correlated with GMP, indicating lower GMP levels in the LM group. As GMP is an important product of purine metabolism, reduced GMP may decrease the energy burden of purine metabolism, allocating more resources to fatty acid and propanoate metabolic pathways [31]. Therefore, F082 in the LM group may enhance overall energy efficiency in yaks by diverting metabolic resources away from GMP production and towards fatty acid and propanoate metabolism.

## CONCLUSION

This study indicates that under low-energy diet conditions, a dietary protein level of 14% is sufficient to meet the growth requirements of yaks. Increasing the protein level beyond 14% does not significantly enhance ADG or slaughter performance and may instead increase metabolic burden. Analysis of the rumen microbiota showed that while protein levels did not significantly influence microbial diversity, the enriched fatty acid and selenocompound metabolism pathways in the LM group could enhance energy storage and utilization efficiency. The increase in Prevotella improved cellulose degradation, boosted VFA production, and enhanced energy supply. Additionally, reduced purine metabolism in the LM group may help lower oxidative stress, thereby allocating more resources to fatty acid and propanoate metabolism. In conclusion, a 14% protein diet not only satisfies the growth requirements of yaks but also optimizes rumen metabolism and microbial community structure, thereby improving energy efficiency. This provides a theoretical basis for optimizing feed formulation and efficient yak feeding under low-energy diet conditions.

## Figures and Tables

**Table 1:** Composition and nutrient levels of the experimental diets (%)

Items	Groups	

	LH	LM
Ingredients		
Corn	1.0	1.0
Wheat	2.8	15.6
Bran	30.3	23.1
Rapeseed meal	19.3	16.6
Soybean meal	7.2	4.5
Limestone	0.6	0.6
CaHPO4	0.4	0.4
NaCl	0.9	0.9
Premix^1)^	2.1	2.1
Oat hay	6.6	1.8
Oat silage	3.8	2.0
Wheat straw	25.0	31.4
Total	100.0	100.0
Nutrient levels^2)^		
ME/(MJ/kg)	9.57	9.55
CP	16.00	14.01
ADF	26.41	26.40
NDF	43.10	42.35
Ca	0.40	0.37
P	0.44	0.39

1) The premix provided the following per kilogram of diets: VA 4,000 IU, VD3 800 TU, VE 40 IU, Cu 15 mg, Fe 600 mg, Zn 30 mg, Mn 40 mg, Se 0.3 mg, I 0.8 mg, Co 0.3 mg.

2) Nutrient levels were measured values (in dry matter).

LH, low-energy high-protein group; LM, low-energy medium-protein group; CP, crude protein; ADF, acid detergent fiber; NDF, neutral detergent fiber; Ca, calcium; P, phosphorus.

**Table 2:** Effects of different protein level diets on growth performance and slaughter performance of yaks

Items	Groups		SEM	p-value

	LM	LH		
IBW/kg	199.75	198.50	0.94	0.537
FBW/kg	261.55	258.80	1.27	0.315
ADG/kg	0.68	0.67	16.98	0.652
CW/kg	183.10	200.40	8.98	0.283
SR/%	55.21	54.84	2.00	0.941

p-values were calculated using independent samples t-tests.

There were no significant differences in ADG, CW, and SR between the LH and LM groups (p>0.05).

LM, low-energy medium-protein group; LH, low-energy high-protein group; SEM, standard error of the mean; IBW, initial body weight; FBW, final body weight; ADG, average daily gain; CW, carcass weight; SR, slaughter rate.

**Table 3:** Effects of different protein level diets on pH, color, shear force and cooking loss of yak *longissimus dorsi* muscle

Items	Groups		SEM	p-value

	LM	LH		
pH 45 min	6.56^a^	5.59^b^	0.17	<0.001
pH 24 h	5.77	5.54	0.08	0.140
L*	27.15^a^	32.43^b^	1.01	0.001
a*	11.48^a^	14.93^b^	0.67	0.002
b*	6.00^a^	9.47^b^	0.65	0.001
Shear force/N	5,206.48	3,266.39	439.12	0.015
Cooking loss/%	39.52	38.65	1.63	0.807

The p-value was determined using an independent samples t-test.

There were significant differences between the LH and LM groups in terms of pH value at 45 minutes, pH value at 24 hours, shear force, and L*, a*, and b* (p<0.05), but no significant differences in cooking loss (p>0.05).

a,b Values within a row with different superscripts differ significantly at p<0.05.

LM, low-energy medium-protein group; LH, low-energy high-protein group; SEM, standard error of the mean.

**Table 4:** Effect of different protein level diets on the sarcoplasm of yak *longissimus dorsi* muscle

Items	Groups		SEM	p-value

	LM	LH		
Hardness/g	3,720.70	3,275.60	218.29	0.34
Elasticity/mm	4.62	4.82	0.11	0.38
Adhesion/g	1,544.44	1,728.40	118.31	0.30
Chewiness/MJ	69.24	61.03	3.66	0.47

The p-value was determined using an independent samples t-test.

There were no significant differences between the LH group and the LM group in terms of hardness, elasticity, adhesiveness, and chewability (p>0.05).

LM, low-energy medium-protein group; LH, low-energy high-protein group; SEM, standard error of the mean.

## Data Availability

The data supporting the findings of this study can be obtained upon reasonable request from the authors. The amplicon sequencing data have been uploaded to the NCBI database and are publicly accessible. The BioProject accession ID is PRJNA1188369.

## SUPPLEMENTARY MATERIAL

**Supplement 1**. Effects of diets with different protein levels on rumen microflora composition of yaks (phylum level).

**Supplement 2**. Effects of diets with different protein levels on rumen microflora composition of yaks (genus level).

**Supplement 3**. Comparison of significantly changed muscle metabolites between the two groups.
