# Supplementary material for: Protein levels alter yak rumen microbiota profiles, meat properties, and longissimus dorsi metabolites
Source: Anim Biosci. 2025 Jul 11;39(5):250027. doi: 10.5713/ab.25.0027 (PMC13153707; doi:10.5713/ab.25.0027)
Supplement: Supplementary file 1 [file ab-25-0027-Supplement-1.pdf]

Supplement 1. Effects of diets with different protein levels on rumen microflora composition of yaks (phylum level)

Items	Groups		SEM	*P*-value
	LM	LH		
Bacteroidota	0.63	0.61	0.02	0.49
Firmicutes	0.30	0.31	0.01	0.65

LM, Low energy medium protein diet; LH, Low energy high protein diet; SEM, standard error mean. The p-value was determined using an independent samples t-test.
