# Supplementary material for: Protein levels alter yak rumen microbiota profiles, meat properties, and longissimus dorsi metabolites
Source: Anim Biosci. 2025 Jul 11;39(5):250027. doi: 10.5713/ab.25.0027 (PMC13153707; doi:10.5713/ab.25.0027)
Supplement: Supplementary file 2 [file ab-25-0027-Supplement-2.pdf]

Supplement 2. Effects of diets with different protein levels on rumen microflora composition of yaks (genus level)

Items	Groups		SEM	*P*-value
	LM	LH		
F082	0.273	0.219	0.017	0.117
Christensenellaceae_R-7_group	0.040	0.041	0.027	0.724
Absconditabacteriales_SR1	0.014	0.017	0.003	0.294
Succiniclasticum	0.028	0.024	0.006	0.734
Rikenellaceae_RC9_gut_group	0.137	0.187	0.013	0.035
Uncultured	0.021	0.026	0.002	0.183
UCG-010	0.013	0.013	0.001	0.993
Muribaculaceae	0.034	0.056	0.012	0.375
UCG-005	0.011	0.011	0.001	0.980
Eubacterium_coprostanoligenes_group	0.009	0.010	0.001	0.477
RF39	0.013	0.015	0.001	0.277
Papillibacter	0.019	0.023	0.002	0.317
Saccharofermentans	0.023	0.016	0.003	0.230
Candidatus_Saccharimonas	0.019	0.018	0.001	0.611
Prevotellaceae_UCG-001	0.036	0.033	0.004	0.692
NK4A214_group	0.018	0.019	0.001	0.521
Prevotellaceae_UCG-003	0.013	0.010	0.001	0.246
Prevotella	0.101	0.053	0.010	0.004

LM, Low energy medium protein diet; LH, Low energy high protein diet; SEM, standard error mean. The p-value was determined using an independent samples t-test.
