# Supplementary material for: Protein levels alter yak rumen microbiota profiles, meat properties, and longissimus dorsi metabolites
Source: Anim Biosci. 2025 Jul 11;39(5):250027. doi: 10.5713/ab.25.0027 (PMC13153707; doi:10.5713/ab.25.0027)
Supplement: Supplementary file 3 [file ab-25-0027-Supplement-3.pdf]

Supplement 3. Comparison of significantly changed muscle metabolites between the two groups

Metabolite names	Groups		SEM	*P*-value
	LM	LH		
Oleamide	2244935790.946	4789111593.896	711169545.066	0.049
8(R)-Hydroxy-(5Z,9E,11Z,14Z)-eicosatetraenoic acid	28367818.743	4217425.204	4972648.762	0.001
N6-Succinyl Adenosine	21566425.451	3906889.970	3850176.850	0.002
Eicosapentaenoic acid	27871646.047	3957619.634	4935115.325	0.001
N-Acetyl-L-methionine	9925980.030	23348309.353	2493497.103	0.001
Uridine 5'-monophosphate	16538930.941	42858915.604	5237518.066	0.019
Methoxyacetyl fentanyl-d5	15474225.745	7961096.311	1552186.020	0.005
Lysops 22:6	18672086.943	31458507.778	3138908.886	0.045
ACar 19:2	5207144.118	1922276.446	782245.505	0.025
N-Acetyl-α-D-glucosamine 1-phosphate	22351390.946	38464559.055	3754848.136	0.049
Uric acid	249904306.904	476195534.395	47854834.373	0.006
cis-5,8,11,14,17-Eicosapentaenoic acid	166431239.762	378849806.597	49976996.721	0.049
2-Ethylhexanoic acid	10829599.335	21187443.769	2407065.843	0.012
Palmitic acid	250411185.691	88392723.242	40983279.822	0.018
ACar 21:1	622625.641	1606730.407	231126.953	0.023
L-(+)-Arabinose	9223398.659	17887791.017	1745844.230	0.005
Methylmalonic acid	172197608.478	385195984.591	45804324.852	0.011
ACar 20:4	349927172.800	74091671.380	54808609.901	0.002
ACar 22:6	8896475.330	768116.904	1538721.129	0.000
Estrone	9787729.601	2969997.950	1189182.156	0.002
ACar 14:1	222781903.598	83423283.502	34987540.562	0.018
L-Cysteine-glutathione gisulfide	7452561.618	19178752.338	2574456.494	0.009
XLR11 N-(2-fluoropentyl) isomer	9508111.918	4902759.971	1001173.941	0.006
SM (d14:0/14:1)	3311731.521	9111195.957	1265647.458	0.035
Valproic acid	79228273.407	7466234.219	14788983.932	0.001

Metabolite names	Groups		SEM	*P*-value
	DZ	DG		
Tetradecanedioic acid	12272044.900	8234437.305	1054757.163	0.035
8Z,11Z,14Z-Eicosatrienoic acid	8887833.153	1926983.947	1689905.095	0.004
LysoPE 18:0	262819708.458	365426537.427	23993928.437	0.037
2-Amino-1,3,4-octadecanetriol	80561280.433	439774726.035	79224779.397	0.002
Hexadecanedioic acid	11215124.681	1529998.788	2508611.000	0.011
L-Argininosuccinate	11852346.371	7253573.113	1011855.608	0.017
Monoolein	75294868.659	443893763.674	75846151.447	0.002
α-Aspartylphenylalanine	4755798.081	8391897.963	836675.044	0.027
2,3,4-Trihydroxybenzoic acid	1092278.180	400542.798	176362.932	0.030
dimethyl 2-(3-nitro-2-pyridyl)malonate	3716599.550	8139666.077	956718.512	0.017
ACar 17:2	7628423.714	2167368.899	1070210.972	0.001
10-Hydroxydecanoic acid	13648142.561	7543617.984	1270047.753	0.006
Uridine5'-Diphospho-N-Acetylgalactosamine	1252215.987	3739592.355	598341.324	0.027
5-Hydroxytryptophan	10177197.357	5570079.873	951100.631	0.006
Succinic acid	2851618879.325	5585595978.079	593560635.115	0.014
o-Toluic Acid	217276276.778	367959479.315	32829186.555	0.010
ACar 17:0	76167207.425	36673634.948	11718170.397	0.041
Uridine monophosphate (UMP)	130205388.022	333289980.435	39544016.624	0.042
2-Hydroxycinnamic acid	7807949.195	12907459.874	1094975.453	0.008
ACar 18:2	200406777.340	36515555.781	31947028.644	0.001
Xylitol	2269577.461	4031027.110	358970.481	0.003
ACar 16:1	6123520.610	1254376.727	962059.946	0.001
N,N-Dimethyldecylamine N-oxide	841839.611	5713168.894	1149033.363	0.005
2-{2-[2,5-di(methoxycarbonyl)anilino]-2-oxoethoxy}acetic acid	5360612.031	3394846.806	525832.433	0.049
Caprolactam	45384002.006	69713456.040	5071164.581	0.014
ACar 15:1	38825106.265	23954810.661	3800526.225	0.047

Metabolite names	Groups		SEM	*P*-value
	LM	LH		
ACar 17:1	253085501.307	33486874.977	47878675.326	0.002
2,6-Dihydroxypurine	3866098.601	9961033.417	1109219.801	0.005
2-Amino-1,3-octadecanediol	26844428.025	212701140.127	35836511.300	0.000
Noroxycodone-d3	3789466.371	173512.734	786833.587	0.003
Cyclic ADP-ribose	109913813.692	52276044.974	15370840.765	0.047
1-(2-furyl)pentane-1,4-dione	3313961.956	5757723.398	600705.381	0.027
ACar 18:3	169445176.449	29796827.591	31190637.132	0.001
ACar 18:1	3815151.275	735133.499	621555.159	0.001
Veratrole	4465776.006	550308.716	868116.484	0.001
4-Hydroxy-2-Oxoglutaric Acid	7651963.317	3447834.527	974197.111	0.010
FAHFA (18:2/20:4)	784053.479	2844894.074	485197.535	0.006
methyl6{[4(trifluoromethyl)anilino]carbonyl}nicotinate	1398343.474	3948843.345	503695.906	0.008
Hexadecanamide	959442052.365	2254718803.684	338813283.592	0.036
Tetrahydrocortisone	1132579.783	399936.134	143663.600	0.003
L-Palmitoylcarnitine	6286910.268	579777.773	1258159.193	0.001
N-Formyl-L-methionine	6177972.618	8388700.321	562704.985	0.042
Cyclopentyl fentanyl-d5	1409269.148	668866.421	169763.106	0.011
3-[(methoxycarbonyl)amino]-2,2,3-trimethylbutanoic acid	183986607.090	223842266.735	9601535.549	0.042
Palmitoylcarnitine cation	88513669.113	28662632.019	15025632.770	0.016
RLK	1498305.334	265029.480	295219.504	0.007
2-{[(4,5-dimethoxy-2-nitrophenethyl)imino]methyl}phenol	225548.394	2373807.844	455207.488	0.001
Adenosine 3'5'-cyclic monophosphate	4654840.134	1499190.649	569530.244	0.000
ACar 19:3	1695858.813	330170.281	300994.487	0.003

Metabolite names	Groups		SEM	*P*-value
	LM	LH		
α -D-Glucose-1,6-bisphosphate	314553785.141	79258797.248	48583169.197	0.019
trans-3-Hexenoic acid	46098834.888	6895199.505	7700832.760	0.001
1-methyl-1H-benzimidazole-2-sulfonic acid	33573091.860	17853493.259	2961731.414	0.005
Flavin adenine dinucleotide (FAD)	61054865.032	45637453.885	3361744.091	0.020
alpha-Farnesene	6964830.672	15254320.964	1846079.896	0.030
Nicotinamide adenine dinucleotide	31864550.828	18011580.615	3201861.921	0.035
S-Adenosylhomocysteine	44449159.212	65998463.984	4589607.548	0.012
Hexanoylcarnitine	883208479.875	2462303366.333	328796392.780	0.003
Esculin	1077626.090	1787996.608	166277.669	0.022
N-(5-Aminopentyl)acetamide	768965.111	1262887.516	120513.267	0.041
N-Arachidonoyl-L-serine	2087698.849	691549.427	480347.782	0.038
3,4-dihydroxy-4-(4-methoxyphenyl)-1,2,3,4-tetrahydroquinolin-2-one	3727900.246	2450433.291	315728.137	0.038
Gamma-Glu-Leu	1737364.180	3494263.863	426596.174	0.030
Tauro-alpha-Muricholic acid sodium salt	1700008.271	460976.975	347741.011	0.026
Xanthosine	36105069.720	101746114.177	11819127.277	0.005
ACar 15:0	81610744.395	36529402.576	10910477.956	0.015
Lipoic acid	106029327.679	55844800.004	11112714.342	0.005
ACar 20:5	113049934.684	15704961.296	20590023.035	0.001
ACar 7:0	42373996.370	81281649.298	9827207.554	0.041
Prostaglandin E3	510550.096	1868526.966	306314.208	0.007
(2S)-4-Oxo-2-phenyl-3,4-dihydro-2H-chromen-7-yl beta-D-glucopyranoside	7051686.887	84912383.989	15038135.373	0.000

Metabolite names	Groups		SEM	*P*-value
	LM	LH		
Dimethylallyl pyrophosphate	3623941.172	5870023.377	483676.4438	0.009
4-Acetamidobutyric Acid	5664560.766	3767773.726	439334.056	0.018
Adenosine triphosphate (ATP)	2966133.595	720975.212	693511.781	0.023
2-(14,15-Epoxyeicosatrienoyl) glycerol	151226.859	643954.438	1266758.978	0.047
Prostaglandin H2	3618822.139	1318039.165	633750.6134	0.024
D-α-Hydroxyglutaric acid	381326909.448	977024520.233	121755592.680	0.005
FAHFA (20:4/3:0)	1657899.400	4383760.977	631175.289	0.042
Isobutyryl carnitine	62008572.123	119240013.203	12947194.893	0.021
Methylmalonate	54501366.395	117558173.723	14518262.582	0.032
Inosine 5'-Monophosphate	957561.990	676603.688	75006.112	0.044
4-Hydroxyisoleucine	54021758.391	39564001.560	3397204.929	0.026
gamma-Glutamyltyrosine	595802.486	1514080.736	217435.818	0.032
L-aspartic Acid	94450171.728	32215106.796	15890705.640	0.026
Boc-beta-cyano-L-alanine	841820.940	2329555.492	326939.299	0.008
4-Methylvaleric Acid	28616705.153	83264822.453	11949185.24	0.006
Guanosine monophosphate (GMP)	24886689.282	106469247.149	14911037.920	0.007
2-Hydroxyisocaproic Acid	27390509.394	58020914.014	7339674.068	0.016
Corey Lactone Diol	69504591.962	120422909.191	11841275.772	0.026
ACar 20:3	45034321.132	10736444.855	7308036.034	0.003
Guanosine monophosphate	37004853.136	100631824.816	13048569.488	0.029
23-Nordeoxycholic acid	5387301.881	55369989.080	12282291.355	0.011
Phenylacetylglycine	97288142.564	61712874.393	8364615.157	0.014
Monobutyl phthalate	104179642.135	47655569.858	10687490.319	0.006
ADP-ribose	43357609.618	73855382.127	8239723.229	0.048
11-Dehydro thromboxane B2	394159.225	1114478.745	163218.949	0.015
Pantetheine	1714179.561	1023025.512	167741.043	0.013
Gly-Tyr-Ala	913398.296	1600553.096	163172.283	0.036
Guanosine	8786448.797	25515534.655	3046180.803	0.015

Metabolite names	Groups		SEM	*P*-value
	LM	LH		
UDP-D-glucuronate	1272788.552	333848.974	226897.671	0.005
Lysopc 17:0	6877457.111	17450826.181	2042446.727	0.024
ACar 22:5	38182327.984	5443737.560	6940883.799	0.001
Deoxycytidine	34511228.192	55584082.370	5303209.022	0.047
4-oxo-4-(4-toluidino)but-2-enoic acid	53332191.828	79391435.224	6657196.670	0.039
Palmitoylcarnitine	1843649597.968	542256527.381	307046940.670	0.006
2-(2-chlorophenyl)-1-cyclohexyl-6-oxopiperidine-3-carboxylic acid	765925.367	1367490.323	127166.626	0.004
2'-Deoxyadenosine	77466065.396	47712674.803	7717746.805	0.041
LPC 16:0	818369024.993	1147227722.578	81951182.809	0.045
N1-(2-chloro-6-fluorobenzyl)-3,4-dimethoxybenzene-1-sulfonamide	876583.338	1583726.215	167011.102	0.034

LM, Low energy medium protein diet; LH, Low energy high protein diet; SEM, standard error mean. The p-value was determined using an independent samples t-test.
